# Integrating network pharmacology and experimental validation to reveal the anti-growth mechanism of panaxadiol against glioblastoma via calcium signaling

**DOI:** 10.3389/fmolb.2025.1598413

**Published:** 2025-05-16

**Authors:** Guobin Qiu, Zhiyong Wu, Dunhui Yang, Luqiu Zhou

**Affiliations:** ^1^ Shenzhen Clinical Medical College, Guangzhou University of Chinese Medicine, Shenzhen, China; ^2^ Department of Neurosurgery, Longgang Central Hospital of Shenzhen, Shenzhen, China

**Keywords:** panaxadiol, GBM, network pharmacology, calcium ions, proliferation

## Abstract

Glioblastoma (GBM) is a highly aggressive brain tumor and is relatively common among malignant brain tumors in adults. Its rapid proliferation and significant invasiveness make its treatment one of the major challenges in brain tumor research. Panaxadiol, a compound extracted from ginseng roots, has been found to have significant therapeutic effects on various types of tumors. Nonetheless, the precise function and underlying mechanisms of this factor in GBM have yet to be thoroughly investigated. In the current study, we employed network pharmacology to explore the potential therapeutic interactions of Panaxadiol within the framework of GBM. Subsequently, we confirmed its efficacy via biological experiments aimed at elucidating the mechanisms through which it exerts its anti-GBM effects. We collected relevant targets of Panaxadiol and differential genes of GBM from multiple databases. The network pharmacology analysis revealed 66 potential targets of Panaxadiol in the context of GBM. Enrichment analysis indicated that these targets might function through several key signaling pathways, including the calcium, cAMP, and cGMP-PKG signaling pathways. Therefore, Panaxadiol may exert its effects by regulating calcium ions. Further, In our study, we employed the MOCDE and CytoHubba plugins within the Cytoscape framework to identify seven hub genes, including GRIA2, GRIN1, GRIN2B, GRM1, GRM5, HTR1A, and HTR2A, and validated their binding capabilities with Panaxadiol through molecular docking. Furthermore, we conducted experiments *in vitro* and *in vivo* experiments, which encompassed CCK-8, colony formation, flow cytometry apoptosis, intracellular calcium ion measurement, and xenograft tumor experiments utilizing nude mice, to validate the function of Panaxadiol in suppressing the growth of GBM via the modulation of calcium ion levels. This study not only revealed the anti-GBM mechanisms of Panaxadiol through network pharmacology but also validated its inhibitory effects on GBM via calcium ion release through *in vitro* and *in vivo* experiments.

## Introduction

GBM is recognized as one of the most common types of brain tumors, which progresses rapidly and is highly invasive, characterized by an unfavorable outcome (Tan et al., 2020). Individuals diagnosed with GBM generally exhibit a median survival time of around 14 months, with the survival rate at the 2-year mark ranging from 20% to 30% (Liu et al., 2023; Rong et al., 2022). Currently, the standard method for managing GBM primarily involves surgical resection, which is often accompanied by radiation therapy, chemotherapy, and immunotherapeutic strategies (Xu et al., 2020). Despite advancements in medical technology, the mortality rate among GBM patients remains high, and survival outcomes are still unsatisfactory (Masui et al., 2017). Therefore, exploring effective therapeutic drugs targeting the molecular mechanisms of GBM is crucial for improving patient prognosis. Panaxadiol is a derivative of triterpenoid saponins that is extracted from the roots of ginseng, characterized by a molecular weight of 460.73 g/mol (Xiao et al., 2017). Panaxadiol exhibits a variety of biological activities, particularly its regulatory effects on intracellular and extracellular calcium ions. It has shown significant roles in anti-inflammatory, anti-tumor, and neuroprotective fields (Li et al., 2016; Liang et al., 2019; Zhu et al., 2017). In terms of anti-inflammation, Panaxadiol mitigates inflammatory responses through the suppression of TNF-α/TNFAR and IL7/IL7R signaling pathways in both macrophages and epithelial cells (Lee et al., 2022). In diabetes, Panaxadiol improves disease conditions by inhibiting the RORγ/IL-17A axis (Tian et al., 2023). It also promotes platelet hemostasis by inducing the release of Ca^2+^. In the field of oncology, Panaxadiol has demonstrated significant anti-tumor activity. For example, it prohibits the progression of pancreatic cancer through the modulation of the JAK2/STAT3 signaling pathway (Fan et al., 2021; Liu et al., 2024) and restricts the progression of colorectal cancer cells through the (HIF)-1α pathway (Wang et al., 2020). These research findings can illustrate the potential of Panaxadiol in cancer therapy. However, its role in GBM is still unclear and requires further exploration of its potential effects. Network pharmacology represents a burgeoning domain that amalgamates insights from bioinformatics, computational science, and pharmacological studies (Hopkins, 2007). It explores the mechanisms of drug action by analyzing the interaction networks between drug and disease targets. Unlike the traditional drug development model that focuses on a single disease, single drug, and single target, network pharmacology adopts a holistic perspective. It illustrates the intricate connections between pharmaceuticals and medical conditions through the lens of a biological network perspective (Nogales et al., 2022; Yan et al., 2024). In this investigation, we utilized network pharmacology to examine the possible therapeutic benefits of Panaxadiol in the context of GBM and to investigate the intricate mechanisms involved. The aim is to promote the application of Panaxadiol in GBM treatment and provide valuable references for clinical therapeutic strategies.

## Materials and methods

### Molecular monomer data

The molecular structure of Panaxadiol was downloaded from the PubChem database, with a Compound CID of 73,498 (Wang et al., 2012).

### Acquisition of panaxadiol-related targets

To obtain target information related to Panaxadiol, we utilized multiple databases for prediction and collection. These databases included the Comparative Toxicogenomics Database (Davis et al., 2023) (CTD, https://ctdbase.org/), SEA Search Server (https://sea.bkslab.org/) (Jin et al., 2022), SwissTargetPrediction (Daina et al., 2019) (http://www.swisstargetprediction.ch/) and TargetNet (Yao et al., 2016) (http://targetnet.scbdd.com/home/index/). During this process, the 2D structure and Isomeric SMILES information of Panaxadiol were obtained from the PubChem database.

### Acquisition of GBM transcriptomic data

We downloaded GBM transcriptomic data in FPKM format from The Cancer Genome Atlas (TCGA, https://portal.gdc.cancer.gov/) (Zhao et al., 2023). The dataset comprised a total of 171 samples derived from tumor tissues, alongside five samples from normal tissues Subsequently, we processed these data using the “limma” R package to prepare for subsequent analyses.

### Differential expression analysis

To obtain differentially expressed genes (DEGs) in the TCGA-GBM cohort, We conducted an analysis of differential gene expression utilizing the “limma” package in R. The screening thresholds were set as |logFC| > 2 and P < 0.05.

### Potential functions of panaxadiol

We utilized the “clusterProfiler” package in R to explore the potential functions of Panaxadiol, focusing on Disease Ontology, Gene Ontology, and the Kyoto Encyclopedia of Genes and Genomes (Kanehisa and Goto, 2000; Schriml et al., 2022).

### PPI network construction

To investigate the potential target network of Panaxadiol in treating GBM, we first used the “VennSchemram” R package to intersect Panaxadiol-related targets with GBM DEGs and visualized the results. Subsequently, We submitted the identified intersecting targets to the STRING database (https://cn.string-db.org/) in order to develop a protein-protein interaction network (Szklarczyk et al., 2025). We chose *Homo sapiens* as the species of interest and established a medium confidence threshold of 0.4 to endure the reliability of the interaction network (Szklarczyk et al., 2021).

### Hub gene screening

We imported the protein-protein interaction network into Cytoscape (v3.10.3) for further analysis and visualization. Using the CytoHubba (Chin et al., 2014) plugin in Cytoscape (Kohl et al., 2011), we performed topological analysis of the network nodes based on algorithms such as MNC, MCC, and Degree. We pinpointed the ten targets that exhibited the highest scores, which were regarded as potential key genes. Degree(Degree Centrality) is an algorithm that assesses the importance of a node by calculating the number of edges directly connected to it. The higher the degree of a node, the more important it is considered to be within the network. MNC (Maximum Neighborhood Component) is an algorithm that evaluates the importance of nodes by identifying key nodes with a larger neighborhood range within the network. MCC (Maximum Clique Centrality) refers to the algorithm that assesses the centrality of nodes by calculating the number of maximum cliques to which a node belongs, thereby identifying nodes that are in key positions within the network.

Then used “VennSchemram” in R to intersect these targets to determine the final hub genes. Additionally, to parse the modular structure the network of interactions between proteins, we employed the MCODE plugin (Bader and Hogue, 2003) with parameters set as degree cutoff = 2, Node Score cutoff = 0.2, K-Core = 2, and Max. Depth = 100 to identify highly interacting subnetwork modules.

### Molecular docking

The protein configurations of the hub genes are available from the PDB database, including GRIA2 (PDB-ID: 2wjx), GRIN1 (PDB-ID: 8vuv), GRIN2B (PDB-ID: 5ewj), GRM1 (PDB-ID: 3 ks9), GRM5 (PDB-ID: 7p2l), HTR1A (PDB-ID: 8pjk), and HTR2A (PDB-ID: 7wc4) (Noguchi and Akiyama, 2003). Subsequently, we performed molecular docking simulations using the 2D structure of Panaxadiol on the CB-Dock2 platform (cadd.labshare.cn) to investigate the binding capabilities of Panaxadiol with these hub genes (Liu et al., 2022).

### Cell culture conditions

U251 cells were purchased from Procell Corp, and U87 cells were sourced from the National Collection of Authenticated Cell Cultures. The cells were cultured in DMEM medium supplemented with 10% fetal bovine serum (FBS). The cells were cultured in an incubator set at 37°C with 5% CO_2_.

### Cell viability assay

The objective of this study is to evaluate the effects of Panaxadiol on the survival rates of the U251 and U87 cell lines. Cells were seeded at a density of approximately 3,000 cells per well in 96-well plates, and were subsequently exposed to various concentrations of Panaxadiol. After a continuous incubation for 48 h, aspirate the culture medium from the wells,and then add the CCK-8 reagent, followed by incubation in the cell culture incubator for approximately 1 h.

### Colony formation assay

The objective of this research is to investigate the effects of extended exposure to Panaxadiol on the growth of GBM cells, U251 and U87 cell lines were maintained in 6-well plates, with a seeding density of 800 cells per well. After 24 h of culture to allow cell attachment, cells were subjected to varying concentrations of Panaxadiol for treatment. Following a culture period of 2 weeks, the cells were subjected to fixation using 4% paraformaldehyde. Subsequently, they were stained with crystal violet to facilitate further observation and quantification.

### EdU assay

We employed the EdU Cell Proliferation Kit labeled to assess the impact of Panaxadiol on the proliferation capabilities of GBM cells. First, Approximately 3 × 10^5^ cells were plated in confocal dishes and seeded under standard conditions for a duration of 24 h. To label proliferating cells, EdU was added to the culture medium for a period of 2 h. Subsequent to the incubation phase, the cells were subject to fixation using a 4% paraformaldehyde solution for a period of 15 min, followed by permeabilization utilizing a 0.3% Triton X-100 solution for a duration of 10 min. After permeabilization, the cells were placed in the Click Reaction Mixture for incubation, which was prepared following the manufacturer’s guidelines, for a duration of 30 min in a dark environment to facilitate the fluorescent labeling of proliferating cells. Following this, the cells were incubated with Hoechst 33,342 dye for a period of 10 min.

### Apoptosis assays

In the apoptosis experiment, we used the Annexin V-FITC/PI Apoptosis Detection Kit (MedChemExpress). Following treatment with Panaxadiol, the cells underwent a washing and digestion process. The resulting cell suspension was then prepared in 195 μL of binding buffer, followed by the addition of 10 μL of Annexin V-FITC and 5 μL of propidium iodide (PI) for staining. The cells were incubated in a dark setting at room temperature for a duration of 15–20 min before analysis was performed using flow cytometry.

### Intracellular Ca^2+^ measurement

We used the Fluo-4 Calcium Assay (Beyotime Biotech) to detect the calcium levels in cells. Following the instructions outlined by the manufacturer, the Fluo-4 Staining Solution was formulated. GBM cells treated with Panaxadiol and seeded in 6-well plates or confocal dishes were washed with PBS. Subsequently, an appropriate volume of Fluo-4 Staining Solution (1 mL for 6-well plates or confocal dishes) was added and incubated at a temperature of 37°C in a dark environment for 30 min. Detection was performed using confocal microscopy and flow cytometry.

### Tumor xenograft experiments

To evaluate the effects of Panaxadiol *in vivo*, we performed tumor xenograft experiments utilizing nude mice that were aged between 4 and 5 weeks. A total of 4 × 10^6^ U87 cells were subcutaneously injected into the mice (n = 5). Mice assigned to the experimental group were administered intraperitoneal injections of Panaxadiol at a dosage of 10 mg/kg every 48 h. Upon completion of the experiment, the mice were humanely euthanized, and the tumors were surgically removed. Tumor volume was calculated using the formula: volume (mm^3^) = width^2^ × length/2. All experimental procedures received approval from the Institutional Animal Care and Use Committee (IACUC) with approval number SUMC 2023-021 and followed the ARRIVE guidelines established by the National Centre for the Replacement, Refinement, and Reduction of Animals in Research (NC3Rs).

### Statistical analysis

In this research, statistical analysis were conducted utilizing GraphPad Prism (v10.1.2) and R software (v4.3.2). For comparisons between two groups, Student’s t-test was used to assess differences; for comparisons involving more than two groups, one-way analysis of variance (ANOVA) was employed to determine differences. The data are expressed as the mean value along with the standard error of the mean (SEM). Every experiment was conducted independently on three separate occasions. A p-value of less than 0.05 was deemed to indicate statistical significance.

## Results

### Acquisition of intersection targets related to panaxadiol and GBM

**FIGURE 1:**
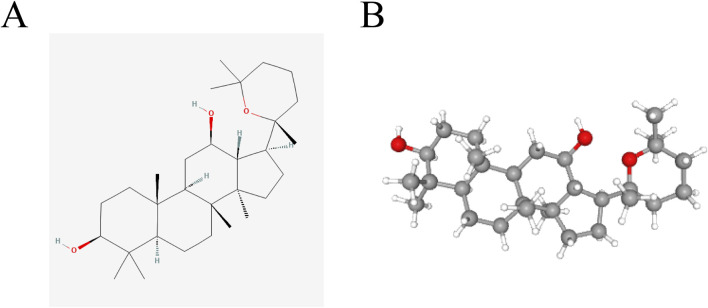
Chemical structures of Panaxadiol: 2D and 3D representations. **(A, B)**.

**FIGURE 2:**
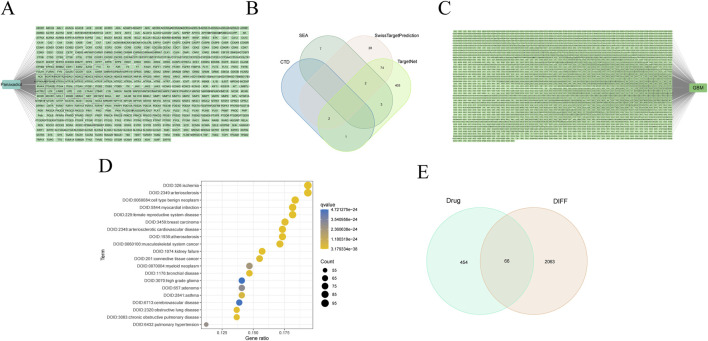
Identification of intersection targets related to Panaxadiol and GBM. **(A, B)** A total of 520 targets related to Panaxadiol were collected from the CTD, SEA, SwissTargetPrediction, and TargetNet databases. **(C)** Differentially expressed genes (DEGs) in the TCGA-GBM cohort. **(D)** Disease Ontology (DO) enrichment analysis of Panaxadiol-related targets. **(E)** Intersection targets between Panaxadiol-related targets and GBM DEGs.

The two-dimensional (2D) and three-dimensional (3D) chemical representations of Panaxadiol are illustrated in (Figures 1A, B), respectively. To obtain targets related to Panaxadiol, we used the CTD, SEA, SwissTargetPrediction, and TargetNet databases to predict and collect its targets, ultimately obtaining a total of 520 related targets (Figures 2A, B; Supplementary Table S1). Subsequently, we downloaded the transcriptomic data of the TCGA-GBM cohort from the TCGA database and conducted differential expression analysis with the “limma” package in R. By setting the threshold to |logFC| > 2 and P < 0.05, we identified 2,129 differentially expressed genes (DEGs) (Figure 2C; Supplementary Table S2). To further explore which diseases the targets of Panaxadiol might be involved in, we performed disease ontology enrichment analysis using these targets (Figure 2D). The results suggest that the targets associated with Panaxadiol are significantly linked to various diseases such as arteriosclerotic cardiovascular disease, musculoskeletal system cancer, and high-grade glioma. Finally, using the “VennSchemram” R package, we intersected the targets of Panaxadiol with the DEGs of GBM, obtaining 66 intersection targets related to both Panaxadiol and GBM (Figure 2E).

### Potential functional roles of panaxadiol and GBM intersection targets

**FIGURE 3:**
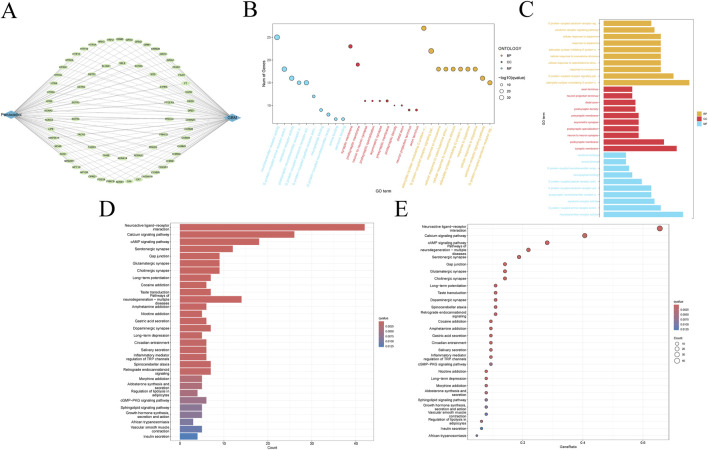
Functional enrichment analysis of intersection targets. **(A)** Network of intersection targets between Panaxadiol-related targets and GBM DEGs. **(B, C)** GO pathway enrichment analysis of the 66 intersection targets. **(D, E)** KEGG pathway enrichment analysis of the 66 intersection targets.

The 66 intersection targets related to Panaxadiol and GBM are shown in (Figure 3A). To investigate the potential functional pathways through which Panaxadiol acts on GBM, We conducted enrichment analyses utilizing GO and KEGG frameworks centered on these identified targets. The GO enrichment is primarily focused on physiological processes, including the signaling pathway of G protein-coupled serotonin receptors, blood vessel diameter maintenance, and phosphatidylinositol phospholipase C activity (Figures 3B, C). Significantly, various pathways associated with calcium were notably highlighted, including the voltage-gated calcium channel complex, calcium channel activity, and the overall calcium channel complex (Supplementary Table S3). This suggests that the therapeutic effect of Panaxadiol on GBM may be mediated through calcium. The findings from the KEGG enrichment analysis lent additional credence to this hypothesis, revealing significant enrichment in the Calcium signaling pathway, the cAMP signaling pathway, and the cGMP-PKG signaling pathway (Figures 3D, E). The Calcium signaling pathway exhibited significant enrichment, aligning with the findings from GO enrichment analysis. Consequently, we hypothesize that Panaxadiol predominantly exerts its effects via the calcium signaling pathway, along with other associated pathways, to modulate the biological behavior of GBM cells.

### Construction of PPI networks and screening of hub genes for intersection targets

**FIGURE 4:**
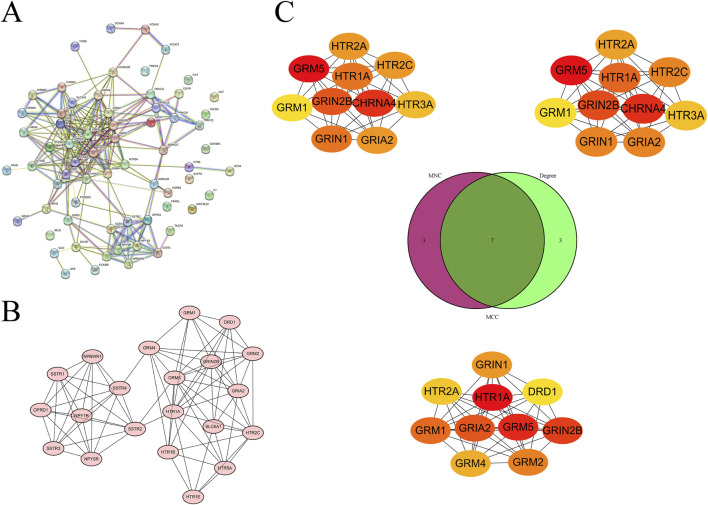
Screening of hub genes from the PPI network of intersection targets. **(A)** PPI network of intersection targets. **(B)** Subnetwork identified by MCODE. **(C)** Hub genes identified by the MNC, MCC, and degree algorithms of CytoHubba, displayed using a Venn diagram.

To further explore the association network among the intersection targets, we successfully constructed and visualized a PPI network of the intersection targets by uploading the 66 targets to the STRING database and using Cytoscape software (Figure 4A). Based on this network, the MCODE plugin was employed to pinpoint the most favorable subnetwork, comprising 21 nodes and 84 edges, which achieved a score of 8.4 (Figure 4B). Subsequently, using the MNC, MCC, and Degree algorithms of the CytoHubba plugin, we screened out the top 10 genes from each algorithm. Through the intersection of the outcomes generated by the three algorithms, we were able to pinpoint the most pivotal hub genes, which were GRIA2, GRIN1, GRIN2B, GRM1, GRM5, HTR1A, and HTR2A (Figure 4C).

### Molecular docking of panaxadiol with hub genes

**FIGURE 5:**
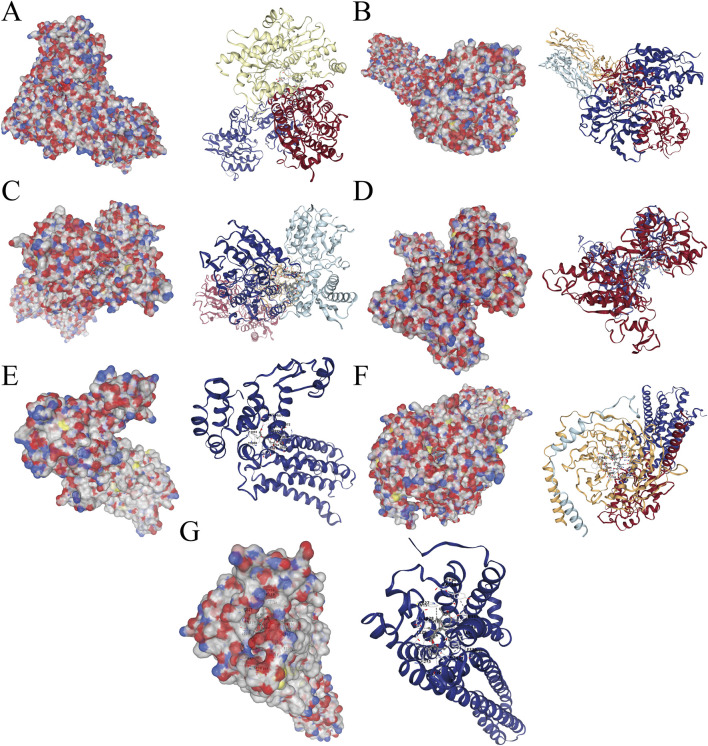
Molecular docking simulations of Panaxadiol with hub genes. **(A)** Panaxadiol-GRIA2. **(B)** Panaxadiol-GRIN1. **(C)** Panaxadiol-GRIN2B. **(D)** Panaxadiol-GRM1. **(E)** Panaxadiol-GRM5. **(F)** Panaxadiol-HTR1A. **(G)** Panaxadiol-HTR2A.

After identifying the hub genes through which Panaxadiol acts on GBM, we used the CB-Dock database to perform molecular docking simulations to assess the binding capabilities of Panaxadiol with these hub genes. The Vina score results were as follows: GRIA2-2wjx: −10.7, GRIN1-8vuv: −9.6, GRIN2B-5ewj: −8.9, GRM1-3 ks9: −9.2, GRM5-7p2l: −8.7, HTR1A-8pjk: −11, and HTR2A-7wc4: −10.1 (Figures 5A–G; Table 1).

**TABLE 1:** Interaction parameters of seven hub targets and panaxadiol.

Protein	PDB ID	CurPocket ID	Vina score
GRIA2	2wjx	C3	−10.7
GRIN1	8vuv	C3	−9.6
GRIN2B	5ewj	C2	−8.9
GRM1	3 ks9	C5	−9.2
GRM5	7p2l	C4	−8.7
HTR1A	8pjk	C2	−11
HTR2A	7wc4	C1	−10.1

### *In Vitro* inhibition of GBM cell proliferation by panaxadiol

**FIGURE 6:**
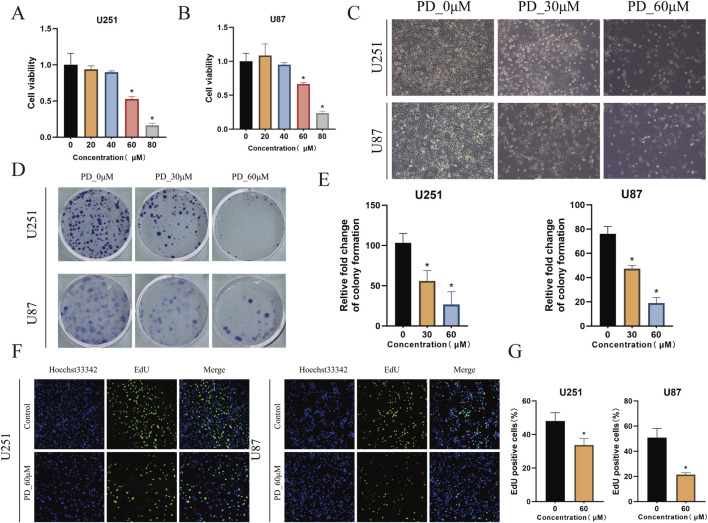
*In vitro* inhibition of GBM cell growth by Panaxadiol. **(A, B)** Effects of Panaxadiol on the viability of U251 and U87 cells. **(C)** Microscopic images of U251 and U87 cells after Panaxadiol treatment. **(D, E)** Representative images of colonies formed by U251 and U87 cells after Panaxadiol treatment. **(F, G)** EdU proliferation assay confirming that Panaxadiol inhibits the proliferation of glioma cells.

To evaluate the therapeutic effects of Panaxadiol on GBM, we investigated its impact on cell proliferation through *in vitro* experiments. The findings indicated that Panaxadiol markedly reduced the viability of GBM cells in a concentration-dependent manner, as demonstrated by the CCK-8 assay (Figures 6A–C). Similarly, in the colony formation assay, the quantity of colonies observed in the Panaxadiol-treated cohort was markedly diminished in comparison to the untreated cohort (Figures 6D, E), indicating that Panaxadiol inhibits the long-term proliferation and clonogenic ability of GBM. Furthermore, the EdU assay demonstrated a notable reduction in the number of EdU-positive cells, suggesting that Panaxadiol significantly inhibits DNA synthesis in GBM cells, thereby impeding their proliferative capacity (Figures 6F, G).

### Panaxadiol inhibits GBM cell proliferation and induces apoptosis via calcium ions in vitro and in vivo models

**FIGURE 7:**
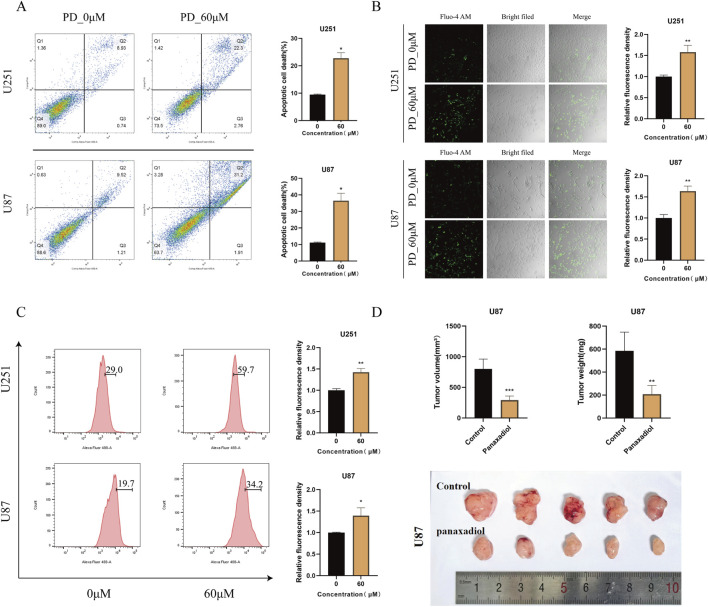
Panaxadiol induces an increase in intracellular calcium ion concentration in GBM,thereby promoting cells apoptosis. **(A)** Flow cytometry experiments confirmed that 60 μM Panaxadiol induced apoptosis in glioma cells after 48 h of treatment. **(B, C)** Confocal and flow cytometry detection of intracellular Ca^2+^ levels after treatment with 60 μM Panaxadiol for 48 h. **(D)** Xenograft tumor experiments in nude mice demonstrated that Panaxadiol significantly inhibited tumor growth in U87 cells.

Flow cytometry apoptosis assays showed that Panaxadiol significantly induces apoptosis in glioma cells. Further experiments indicated that Panaxadiol inhibits GBM cell proliferation and induces apoptosis by increasing intracellular calcium ion influx (Figure 7A). Intracellular calcium ion levels were detected using flow cytometry and confocal microscopy with Fluo-4 staining. The findings indicated that the intensity of green fluorescence was markedly increased in the group treated with Panaxadiol when compared to the control group, indicating a significant increase in intracellular calcium ion levels (Figures 7B, C). Additionally, we validated the results of network pharmacology and *in vitro* cell experiments through a U87 cell xenograft tumor experiment in nude mice. *In vivo* experiments, Panaxadiol was administered via intraperitoneal injection in mice to evaluate its effects on tumor progression. The findings indicated that Panaxadiol exhibited a substantial inhibitory effect on tumor proliferation within the U87 xenograft model. The tumor volume and weight of the excised tumors are shown in (Figure 7D), further confirming the antitumor effects of Panaxadiol.

## Discussion

GBM is a relatively common and highly malignant tumor of the central nervous system. Its tumor cells exhibit high pleomorphism, frequent mitotic figures, and are often accompanied by necrosis (Schaff and Mellinghoff, 2023). The outlook for patients diagnosed with GBM is particularly dismal, with median survival rates estimated to be between 12 and 18 months (Rong et al., 2022). Patients who do not receive any treatment typically survive for only about 3 months. Despite advancements in medical technology, which have extended the survival period for some treated patients to 8–18 months, the overall survival situation remains grim. At present, the available treatment modalities for GBM consist of surgical removal, chemotherapy, radiation therapy, and immunotherapy (Xu et al., 2020; Zhang et al., 2025). Surgical resection is the first step in the comprehensive treatment of GBM. However, due to the special location of the tumor, complete resection is rarely achieved. This not only leads to frequent tumor recurrence but also causes severe postoperative complications (Kirkpatrick and Sampson, 2014). Consequently, it is essential to develop some therapeutic methods from the molecular mechanism level to enhance the outcomes for patients diagnosed with GBM.

Panaxadiol is a compound extracted and isolated mainly from plants in the genus Panax, such as ginseng, American ginseng, and notoginseng. Its molecular formula is C_30_H_52_O_3_, with a molecular weight of 460.73 g/mol (Xiao et al., 2017). Panaxadiol plays important roles in multiple key areas, including anti-tumor, cardiovascular protection, neuroprotection, antioxidant, and immune regulation. For example, Panaxadiol can prevent LPS-induced cardiotoxicity by improving mitochondrial function (Yang et al., 2025). Additionally, it has the potential to enhance the management of obesity by facilitating the transformation of white adipose tissue into beige adipose tissue (Lv et al., 2023). In terms of tumor therapy, Panaxadiol has demonstrated a synergistic effect when association with irinotecan in the management of colon cancer, facilitating programmed cell death in colon cancer (Du et al., 2012). It significantly influences the decrease in both the growth and movement of breast cancer cells (Shao et al., 2024; Xu et al., 2021). However, the functions of Panaxadiol in GBM remain to be fully understood and require further exploration and research. Therefore, this research employed a network pharmacology methodology alongside experimental validation to explore the possible mechanisms through which Panaxadiol may exert therapeutic effects in the treatment of GBM, aiming to provide a new perspective and theoretical basis for the selection and application of drugs in GBM treatment.

We first screened and collected 520 targets related to Panaxadiol from the CTD, SEA, SwissTargetPrediction, and TargetNet databases. Based on these targets, we performed DO enrichment analysis. As expected, The findings indicated that these targets exhibited significant enrichment within categories including arteriosclerotic cardiovascular disease, musculoskeletal system cancer, and high-grade glioma, indicating a close relationship between Panaxadiol-related targets and GBM. Subsequently, we downloaded the TCGA-GBM transcriptomic data from the TCGA database and obtained 2,129 DEGs after differential expression analysis. To further clarify the specific targets through which Panaxadiol acts in GBM, we intersected the previously obtained Panaxadiol-related targets with the GBM DEGs, ultimately identifying 66 related intersection targets.

To explore the potential functional roles of these 66 intersection targets in the management of GBM by Panaxadiol, we conducted enrichment analyses for GO and KEGG. The findings indicated a noteworthy correlation between these targets and various signaling pathways, including Calcium channel activity, the Calcium channel complex, and the Calcium signaling pathway. Calcium functions as a vital second messenger within the body, significantly influencing intracellular signaling pathways. It is significantly involved in the regulation of various biological processes, such as cell growth, movement, and programmed cell death (Patergnani et al., 2020). The abnormal modulation of calcium signaling, whether through its activation or inhibition, is intricately associated with the initiation, advancement, and spread of tumors (Wu et al., 2021). For example, Rutin possesses the capability to impede the growth and dissemination of murine breast cancer cells by influencing the calcium signaling pathway (Li et al., 2021). Additionally, NFATC3, which is related to the Calcium signaling pathway, is involved in alcohol-induced breast cancer growth (Ho and Lin, 2021). In GBM-related studies, Ca^2+^ is considered an important regulator of GBM tumorigenesis (Afshari et al., 2020). Consequently, we hypothesize that Panaxadiol could apply its anti-GBM effects by modulating Ca^2+^ via these intersecting targets.

To further identify the key core targets among these intersection targets, we used the MNC, MCC, and Degree algorithms of the CytoHubba plugin for screening. We ultimately identified core targets including GRIA2, GRIN1, GRIN2B, GRM1, GRM5, HTR1A, and HTR2A. These fundamental targets are crucial in the initiation and progression of tumors. For example, GRIA2 is considered an important gene related to colon cancer staging (Chen et al., 2023). GRIN1 can act as a key gene in the neuro-related classification of endometrial cancer and is associated with calcium signaling (Chen et al., 2022). In colon cancer, GRIN2B is related to colon cancer and the endoplasmic reticulum response (Yuan et al., 2024). GRM1 can serve as a histological marker for distinguishing chondromyxoid fibroma (Toland et al., 2022). GRM5 is associated with breast cancer therapy (Qayoom et al., 2023). HTR1A has the potential to impede the advancement of triple-negative breast cancer via the TGF-β signaling pathway (Liu et al., 2022). HTR2A influences patient prognosis in gliomas through immune regulation (Shen et al., 2023). Meanwhile, we used the MCODE plugin to identify a relevant subnetwork. The fact that some nodes in this subnetwork are consistent with the core targets previously screened out further underscores the importance of these targets. To assess the binding affinity of these primary targets with Panaxadiol, we performed molecular docking simulations using the CB-Dock2 database. The findings indicated that the core targets exhibited comparatively strong binding affinities with Panaxadiol, which will be conducive to the development of Panaxadiol-based drugs and the optimization of their therapeutic effects.

To comprehensively evaluate the therapeutic effects of Panaxadiol on GBM, we performed both *in vitro* and *in vivo* experiments utilizing two GBM cell lines, U251 and U87. *In vitro* studies comprised cell viability assessments, colony formation analyses, and EdU incorporation assays to evaluate the impact of Panaxadiol on the proliferative potential of GBM cells. The results demonstrated that Panaxadiol significantly inhibited the proliferation of GBM cells in a dose-dependent manner. Additionally, apoptosis assays revealed that Panaxadiol could effectively induce apoptosis in GBM cells, further confirming its potential anti-tumor activity. To investigate the mechanisms that contribute to the anti-tumor properties of Panaxadiol, we conducted intracellular Ca^2+^ measurements. Fluo-4, a dynamic single-wavelength fluorescent Ca^2+^ indicator, reflects increased cytoplasmic Ca^2+^ levels through enhanced fluorescence intensity (Gee et al., 2000). The findings indicated that the application of Panaxadiol resulted in a notable increased of Ca^2+^ levels within GBM cells, leading calcium overload and the GBM cells to apoptosis (Liu et al., 2015). This result is consistent with our earlier hypothesis, indicating that Panaxadiol exerts its anti-tumor effects by regulating calcium ion levels and associated signaling pathways, thereby inhibiting GBM growth and inducing apoptosis. To provide additional confirmation regarding the therapeutic benefits of Panaxadiol, we conducted *in vivo* experiments using a xenograft tumor model in nude mice. The analysis of xenograft tumors demonstrated that both the volume and weight of tumors in the Panaxadiol-treated cohort were significantly reduced when contrasted with the control group. This outcome aligns with the observations derived from *in vitro* experiments, confirming at the *in vivo* level that Panaxadiol inhibits GBM proliferation and induces apoptosis by mediating cytoplasmic Ca^2+^ concentration and related signaling pathways.

## Conclusion

In the present research, we utilized a comprehensive strategy that integrates network pharmacology, molecular docking techniques, and an assortment of both *in vitro* and *in vivo* experiments. This approach allowed us to thoroughly substantiate that Panaxadiol inhibits the growth of GBM and enhances apoptotic processes by influencing the levels of cytoplasmic Ca^2+^ concentrations. This finding not only demonstrates the great potential of Panaxadiol in GBM treatment but also provides a new perspective for research in this field, promoting the clinical utilization of medications for GBM while simultaneously laying a solid foundation for prospective basic research.

## Data Availability

The datasets presented in this study can be found in online repositories. The names of the repository/repositories and accession number(s) can be found in the article/Supplementary Material.

## References

[B1] Afshari A. R. Mollazadeh H. Soukhtanloo M. Hosseini A. Mohtashami E. Jalili-Nik M. (2020). Modulation of calcium signaling in glioblastoma multiforme: a therapeutic promise for natural products. Mini Rev. Med. Chem. 20, 1879–1899. 10.2174/1389557520666200807133659 32767939

[B2] Bader G. D. Hogue C. W. V. (2003). An automated method for finding molecular complexes in large protein interaction networks. Bmc Bioinforma. 4, 2. 10.1186/1471-2105-4-2 PMC149346 12525261

[B3] Chen B. Chakrobortty N. Saha A. K. Shang X. (2023). Identifying colon cancer stage related genes and their cellular pathways. Front. Genet. 14, 1120185. 10.3389/fgene.2023.1120185 36741325 PMC9893497

[B4] Chen F. Qin T. Zhang Y. Wei L. Dang Y. Liu P. (2022). Reclassification of endometrial cancer and identification of key genes based on neural-related genes. Front. Oncol. 12, 951437. 10.3389/fonc.2022.951437 36212450 PMC9537575

[B5] Chin C. Chen S. Wu H. Ho C. Ko M. Lin C. (2014). Cytohubba: identifying hub objects and sub-networks from complex interactome. Bmc Syst. Biol. 8 (Suppl. 4), S11. 10.1186/1752-0509-8-S4-S11 25521941 PMC4290687

[B6] Daina A. Michielin O. Zoete V. (2019). Swisstargetprediction: updated data and new features for efficient prediction of protein targets of small molecules. Nucleic Acids Res. 47, W357-W364–W364. 10.1093/nar/gkz382 31106366 PMC6602486

[B7] Davis A. P. Wiegers T. C. Johnson R. J. Sciaky D. Wiegers J. Mattingly C. J. (2023). Comparative toxicogenomics database (ctd): update 2023. Nucleic Acids Res. 51, D1257–D1262. 10.1093/nar/gkac833 36169237 PMC9825590

[B8] Du G. Wang C. Zhang Z. Wen X. Somogyi J. Calway T. (2012). Caspase-mediated pro-apoptotic interaction of panaxadiol and irinotecan in human colorectal cancer cells. J. Pharm. Pharmacol. 64, 727–734. 10.1111/j.2042-7158.2012.01463.x 22471369 PMC3349342

[B9] Fan X. Fu H. Xie N. Guo H. Fu T. Shan Y. (2021). Inhibition of jak2/stat3 signaling pathway by panaxadiol limits the progression of pancreatic cancer. Aging (Albany Ny) 13, 22830–22842. 10.18632/aging.203575 34623971 PMC8544303

[B10] Gee K. R. Brown K. A. Chen W. N. Bishop-Stewart J. Gray D. Johnson I. (2000). Chemical and physiological characterization of fluo-4 ca(2+)-indicator dyes. Cell. Calcium 27, 97–106. 10.1054/ceca.1999.0095 10756976

[B11] Ho C. Lin C. (2021). Genes associated with calcium signaling are involved in alcohol-induced breast cancer growth. Alcohol Clin. Exp. Res. 45, 79–91. 10.1111/acer.14521 33222221

[B12] Hopkins A. L. (2007). Network pharmacology. Nat. Biotechnol. 25, 1110–1111. 10.1038/nbt1007-1110 17921993

[B13] Jin Z. Zhao H. Luo Y. Li X. Cui J. Yan J. (2022). Identification of core genes associated with the anti-atherosclerotic effects of salvianolic acid b and immune cell infiltration characteristics using bioinformatics analysis. Bmc Complement. Med. Ther. 22, 190. 10.1186/s12906-022-03670-6 35842645 PMC9288713

[B14] Kanehisa M. Goto S. (2000). Kegg: kyoto encyclopedia of genes and genomes. Nucleic Acids Res. 28, 27–30. 10.1093/nar/28.1.27 10592173 PMC102409

[B15] Kirkpatrick J. P. Sampson J. H. (2014). Recurrent malignant gliomas. Semin. Radiat. Oncol. 24, 289–298. 10.1016/j.semradonc.2014.06.006 25219814 PMC4522935

[B16] Kohl M. Wiese S. Warscheid B. (2011). Cytoscape: software for visualization and analysis of biological networks. Methods Mol. Biol. 696, 291–303. 10.1007/978-1-60761-987-1_18 21063955

[B17] Lee J. Yang Y. Tao Y. Yi Y. Cho J. Y. (2022). Korean Red Ginseng saponin fraction exerts anti-inflammatory effects by targeting the NF-κB and AP-1 pathways. J. Ginseng Res. 46, 489–495. 10.1016/j.jgr.2022.02.004 35600780 PMC9120761

[B18] Li H. Kang T. Qi B. Kong L. Jiao Y. Cao Y. (2016). Neuroprotective effects of ginseng protein on pi3k/akt signaling pathway in the hippocampus of d-galactose/alcl3 inducing rats model of alzheimer's disease. J. Ethnopharmacol. 179, 162–169. 10.1016/j.jep.2015.12.020 26721223

[B19] Li Q. Xu D. Gu Z. Li T. Huang P. Ren L. (2021). Rutin restrains the growth and metastasis of mouse breast cancer cells by regulating the microrna-129-1-3p-mediated calcium signaling pathway. J. Biochem. Mol. Toxicol. 35, e22794. 10.1002/jbt.22794 33913213

[B20] Liang X. Yao Y. Lin Y. Kong L. Xiao H. Shi Y. (2019). Panaxadiol inhibits synaptic dysfunction in alzheimer's disease and targets the fyn protein in app/ps1 mice and app-sh-sy5y cells. Life Sci. 221, 35–46. 10.1016/j.lfs.2019.02.012 30735733

[B21] Liu A. Jiang B. Song C. Zhong Q. Mo Y. Yang R. (2023). Isoliquiritigenin inhibits circ0030018 to suppress glioma tumorigenesis via the mir-1236/her2 signaling pathway. Medcomm 4 (2020), e282. 10.1002/mco2.282 37250146 PMC10220153

[B22] Liu F. Gao A. Zhang M. Li Y. Zhang F. Herman J. G. (2024). Methylation of fam110c is a synthetic lethal marker for atr/chk1 inhibitors in pancreatic cancer. J. Transl. Int. Med. 12, 274–287. 10.2478/jtim-2023-0128 39081276 PMC11284899

[B23] Liu K. Yang S. Lin Y. Lin J. Lee Y. Wang J. (2015). Fluoxetine, an antidepressant, suppresses glioblastoma by evoking ampar-mediated calcium-dependent apoptosis. Oncotarget 6, 5088–5101. 10.18632/oncotarget.3243 25671301 PMC4467135

[B24] Liu Q. Sun H. Liu Y. Li X. Xu B. Li L. (2022). Htr1a inhibits the progression of triple-negative breast cancer via tgf-beta canonical and noncanonical pathways. Adv. Sci. (Weinh) 9, e2105672. 10.1002/advs.202105672 35199941 PMC9036047

[B25] Liu Y. Yang X. Gan J. Chen S. Xiao Z. Cao Y. (2022). Cb-dock2: improved protein-ligand blind docking by integrating cavity detection, docking and homologous template fitting. Nucleic Acids Res. 50, W159–W164. 10.1093/nar/gkac394 35609983 PMC9252749

[B26] Lv Y. Lv X. Feng J. Cheng F. Yu Z. Guan F. (2023). (20r)-panaxadiol improves obesity by promoting white fat beigeing. Front. Pharmacol. 14, 1071516. 10.3389/fphar.2023.1071516 36909162 PMC9992182

[B27] Masui K. Kato Y. Sawada T. Mischel P. S. Shibata N. (2017). Molecular and genetic determinants of glioma cell invasion. Int. J. Mol. Sci. 18, 2609. 10.3390/ijms18122609 29207533 PMC5751212

[B28] Nogales C. Mamdouh Z. M. List M. Kiel C. Casas A. I. Schmidt H. H. H. W. (2022). Network pharmacology: curing causal mechanisms instead of treating symptoms. Trends Pharmacol. Sci. 43, 136–150. 10.1016/j.tips.2021.11.004 34895945

[B29] Noguchi T. Akiyama Y. (2003). Pdb-reprdb: a database of representative protein chains from the protein data bank (pdb) in 2003. Nucleic Acids Res. 31, 492–493. 10.1093/nar/gkg022 12520060 PMC165469

[B30] Patergnani S. Danese A. Bouhamida E. Aguiari G. Previati M. Pinton P. (2020). Various aspects of calcium signaling in the regulation of apoptosis, autophagy, cell proliferation, and cancer. Int. J. Mol. Sci. 21, 8323. 10.3390/ijms21218323 33171939 PMC7664196

[B31] Qayoom H. Alshehri B. Ul Haq B. Almilaibary A. Alkhanani M. Ahmad Mir M. (2023). Decoding the molecular mechanism of stypoldione against breast cancer through network pharmacology and experimental validation. Saudi J. Biol. Sci. 30, 103848. 10.1016/j.sjbs.2023.103848 37964781 PMC10641555

[B32] Rong L. Li N. Zhang Z. (2022). Emerging therapies for glioblastoma: current state and future directions. J. Exp. Clin. Cancer Res. 41, 142. 10.1186/s13046-022-02349-7 35428347 PMC9013078

[B33] Schaff L. R. Mellinghoff I. K. (2023). Glioblastoma and other primary brain malignancies in adults: a review. Jama 329, 574–587. 10.1001/jama.2023.0023 36809318 PMC11445779

[B34] Schriml L. M. Munro J. B. Schor M. Olley D. McCracken C. Felix V. (2022). The human disease ontology 2022 update. Nucleic Acids Res. 50, D1255–D1261. 10.1093/nar/gkab1063 34755882 PMC8728220

[B35] Shao X. Xie N. Chen Z. Wang X. Cao W. Zheng Y. (2024). Inetetamab for injection in combination with vinorelbine weekly or every three weeks in her2-positive metastatic breast cancer: a multicenter, randomized, phase ii clinical trial. J. Transl. Int. Med. 12, 466–477. 10.1515/jtim-2024-0022 39513033 PMC11538898

[B36] Shen J. Wang Q. Lu F. Xu H. Wang P. Feng Y. (2023). Prognostic and immunomodulatory roles of schizophrenia-associated genes htr2a, comt, and prodh in pan-cancer analysis and glioma survival prediction model. Front. Immunol. 14, 1201252. 10.3389/fimmu.2023.1201252 37564635 PMC10411190

[B37] Szklarczyk D. Gable A. L. Nastou K. C. Lyon D. Kirsch R. Pyysalo S. (2021). The string database in 2021: customizable protein-protein networks, and functional characterization of user-uploaded gene/measurement sets. Nucleic Acids Res. 49, D605–D612. 10.1093/nar/gkaa1074 33237311 PMC7779004

[B38] Szklarczyk D. Nastou K. Koutrouli M. Kirsch R. Mehryary F. Hachilif R. (2025). The string database in 2025: protein networks with directionality of regulation. Nucleic Acids Res. 53, D730–D737. 10.1093/nar/gkae1113 39558183 PMC11701646

[B39] Tan A. C. Ashley D. M. Lopez G. Y. Malinzak M. Friedman H. S. Khasraw M. (2020). Management of glioblastoma: state of the art and future directions. Ca Cancer J. Clin. 70, 299–312. 10.3322/caac.21613 32478924

[B40] Tian S. Chen S. Feng Y. He J. Li Y. (2023). Ginseng-derived panaxadiol ameliorates STZ-induced type 1 diabetes through inhibiting RORγ/IL-17A axis. Acta Pharmacol. Sin. 44, 1217–1226. 10.1038/s41401-022-01042-x 36650291 PMC10203104

[B41] Toland A. M. S. Lam S. W. Varma S. Wang A. Howitt B. E. Kunder C. A. (2022). Grm1 immunohistochemistry distinguishes chondromyxoid fibroma from its histologic mimics. Am. J. Surg. Pathol. 46, 1407–1414. 10.1097/PAS.0000000000001921 35650682 PMC9481662

[B42] Wang Y. Xiao J. Suzek T. O. Zhang J. Wang J. Zhou Z. (2012). Pubchem's bioassay database. Nucleic Acids Res. 40, D400–D412. 10.1093/nar/gkr1132 22140110 PMC3245056

[B43] Wang Z. Li M. Y. Zhang Z. H. Zuo H. X. Wang J. Y. Xing Y. (2020). Panaxadiol inhibits programmed cell death-ligand 1 expression and tumour proliferation via hypoxia-inducible factor (HIF)-1α and STAT3 in human colon cancer cells. Pharmacol. Res. 155, 104727. 10.1016/j.phrs.2020.104727 32113874

[B44] Wu L. Lian W. Zhao L. (2021). Calcium signaling in cancer progression and therapy. Febs J. 288, 6187–6205. 10.1111/febs.16133 34288422

[B45] Xiao S. Chen S. Sun Y. Zhou W. Piao H. Zhao Y. (2017). Synthesis and anti-tumor evaluation of panaxadiol halogen-derivatives. Bioorg Med. Chem. Lett. 27, 4204–4211. 10.1016/j.bmcl.2017.06.061 28757064

[B46] Xu L. Zhang X. Xiao S. Li X. Jiang H. Wang Z. (2021). Panaxadiol as a major metabolite of ad-1 can significantly inhibit the proliferation and migration of breast cancer cells: *in vitro* and *in vivo* study. Bioorg Chem. 116, 105392. 10.1016/j.bioorg.2021.105392 34619469

[B47] Xu S. Tang L. Li X. Fan F. Liu Z. (2020). Immunotherapy for glioma: current management and future application. Cancer Lett. 476, 1–12. 10.1016/j.canlet.2020.02.002 32044356

[B48] Yan J. Sun H. Xin X. Huang T. (2024). Association and mechanism of montelukast on depression: a combination of clinical and network pharmacology study. J. Affect Disord. 360, 214–220. 10.1016/j.jad.2024.05.130 38824963

[B49] Yang Z. Gao Y. Li D. Zhao L. Du Y. (2025). Panaxadiol saponin alleviates lps-induced cardiomyopathy similar to dexamethasone via improving mitochondrial quality control. Shock 63, 282–291. 10.1097/SHK.0000000000002449 39178130 PMC11776890

[B50] Yao Z. Dong J. Che Y. Zhu M. Wen M. Wang N. (2016). Targetnet: a web service for predicting potential drug-target interaction profiling via multi-target sar models. J. Comput. Aided Mol. Des. 30, 413–424. 10.1007/s10822-016-9915-2 27167132

[B51] Yuan Z. Wang Y. Xu S. Zhang M. Tang J. (2024). Construction of a prognostic model for colon cancer by combining endoplasmic reticulum stress responsive genes. J. Proteomics 309, 105284. 10.1016/j.jprot.2024.105284 39159861

[B52] Zhang X. Zhang X. Zhu J. Yi Z. Cao H. Tang H. (2025). An mri radiogenomic signature to characterize the transcriptional heterogeneity associated with prognosis and biological functions in glioblastoma. Front. Biosci. Landmark Ed. 30, 36348. 10.31083/FBL36348 40152396

[B53] Zhao S. Chi H. Yang Q. Chen S. Wu C. Lai G. (2023). Identification and validation of neurotrophic factor-related gene signatures in glioblastoma and Parkinson's disease. Front. Immunol. 14, 1090040. 10.3389/fimmu.2023.1090040 36825022 PMC9941742

[B54] Zhu J. Liang Y. Yue S. Fan G. Zhang H. Zhang M. (2017). Combination of panaxadiol and panaxatriol type saponins and ophioponins from shenmai formula attenuates lipopolysaccharide-induced inflammatory injury in cardiac microvascular endothelial cells by blocking nf-kappa b pathway. J. Cardiovasc Pharmacol. 69, 140–146. 10.1097/FJC.0000000000000450 28266999

